# Action potential repolarization enabled by Ca^++ ^channel deactivation in PSpice simulation of smooth muscle propagation

**DOI:** 10.1186/1475-925X-4-71

**Published:** 2005-12-30

**Authors:** Lakshminarayanan Ramasamy, Nicholas Sperelakis

**Affiliations:** 1 Dept. of Electrical Computer Engineering and Computer Science University of Cincinnati College of Engineering Cincinnati, OH 45219 USA; 2 Dept. of Molecular & Cellular Physiology University of Cincinnati College of Medicine Cincinnati, OH 45267-0576 USA; 3 Dept. of Molecular & Cellular Physiology University of Cincinnati College of Medicine Cincinnati, OH 45267-0576 USA

## Abstract

**Background:**

Previously, only the rising phase of the action potential (AP) in cardiac muscle and smooth muscle could be simulated due to the instability of PSpice upon insertion of a second black box (BB) into the K^+ ^leg of the basic membrane unit. This restriction was acceptable because only the transmission of excitation from one cell to the next was investigated.

**Methods:**

In the current work, the repolarization of the AP was accomplished by inserting a second BB into the Ca^++ ^leg of the basic membrane unit. Repolarization of the AP was produced, not through an activation of the K^+ ^channel conductance, but rather through a mimicking of the deactivation of the Ca^++ ^channel conductance. Propagation of complete APs was studied in a chain (strand) of 10 smooth muscle cells, in which various numbers of gap-junction (gj) channels (assumed to be 100 pS each) were inserted across the cell junctions.

**Results:**

The shunt resistance across the junctions produced by the gj-channels (R_gj_) was varied from 100,000 MΩ (0 gj-channels) to 10,000 MΩ (1 gj-channel), to 1,000 MΩ (10 channels), to 100 MΩ (100 channels), to 10 MΩ (1000 channels), and to 1.0 MΩ (10,000 channels). Velocity of propagation (θ, in cm/sec) was calculated from the measured total propagation time (TPT, the time difference between when the AP rising phase of the first cell and the last cell crossed -20 mV), assuming a constant cell length of 200 μm. When there were no gj-channels, or only one, the transmission of excitation between cells was produced by the electric field (EF), i.e., the negative junctional cleft potential, that is generated in the narrow junctional clefts (e.g., 100 A) when the prejunctional membrane fires an AP (a fraction of a millisecond before the adjacent surface membrane). There were significant end-effects at the termination of the strand, such that the last cell (cell #10) failed to fire, or fired after a prolonged delay. This end-effect was abolished when the strand termination resistance (R_bt_) was increased from 1.0 KΩ to 600 MΩ. When there were 1000 or 10,000 gj-channels, the transmission of excitation was produced by local-circuit current flow from one cell to the next through the gj-channels.

**Discussion:**

In summary, it is now possible to simulate complete APs in smooth muscle cells that could propagate along a single chain of 10 cells, even when there were no gj-channels between the cells.

## Introduction

There are no low-resistance connections between the cells in several different cardiac muscle and smooth muscle preparations [1-3]. In a computer simulation study of propagation in cardiac muscle, firing of the pre-junctional membrane generated an electric field (EF) in the narrow junctional clefts, which depolarized the postjunctional membrane to its threshold [4-6]. This causes excitation of the postjunctional cell, after a brief junctional delay. The total propagation time consists primarily of the summed junctional delays. This results in a staircase-shaped propagation, the surface sarcolemma of each cell firing almost simultaneously [5]. Propagation has been demonstrated to be discontinuous (or saltatory) in cardiac muscle [7-10]. A high density of fast Na^+ ^channels are localized in the junctional membranes of the intercalated disks of cardiac muscle [6,11,12], consistent with the requirement of the junctional membrane being more excitable than the surface membrane (for the EF mechanism to work [3-6]). In connexin-43 and Cx40 knockout mice, propagation in the heart still occurs, but it is slowed [13-16], as predicted by the previous PSpice simulation studies. Propagation by mechanisms not requiring low-resistance connections have also been proposed by others [6,8,9].

Propagation of APs in cardiac muscle and smooth muscle were previously modeled using the PSpice program for circuit design and analysis. The studies corroborated the earlier reports that the EF developed in the junctional cleft is sufficiently large to allow the transfer of excitation without the requirement for a gap junction [17-19]. In those studies, a second black-box (BB-2) could not be inserted into the K^+ ^leg of the Hodgkin-Huxley circuit of the basic membrane units because that caused the PSpice program to become very erratic and unstable. Therefore, only the rising phase of the APs was simulated. This defect in the program was acceptable, since only the mechanism(s) for the transfer of excitation from one cell to the next was under investigation. For this purpose, only the fast rising phase of the AP was necessary. PSpice has recently used by others to model conduction in nerve fibers [20].

The purpose of the present study was to determine whether another method for repolarization could be devised that would circumvent the instability problem. So it was decided to try inserting a BB-2 into the same leg as the first BB (BB-1), namely into the Ca^++ ^leg, to mimic the deactivation of the Ca^++ ^channel, and thereby produce repolarization of the AP. This method worked. Thus, repolarization was produced, not by activation of the K^+ ^conductance, but instead by deactivation of the Ca^++^conductance. Similar results were found for cardiac muscle simulations [21].

## Methods

**Figure 1:**
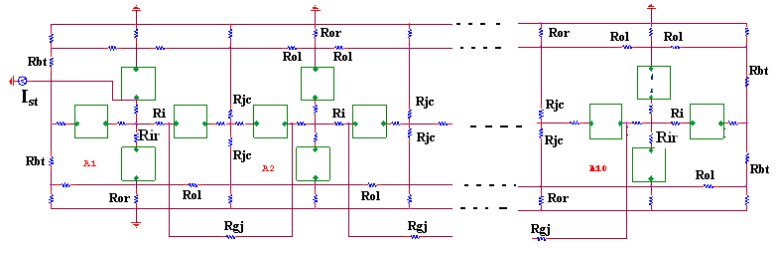
Circuit diagram for the single chain of ten smooth muscle cells bathed in a bath of Ringer solution. Only the two ends of the strand are depicted so that the circuit components can be seen more clearly. Electrical stimulation (0.25 nA, 0.5 ms rectangular current pulses) was applied to the inside of the first cell (cell #1). The AP propagated from the stimulated cell #1 down the entire chain. A variable shunt resistance (R_gj_) was inserted across each of the nine cell junctions to reflect various numbers of gap-junction channels (0, 1, 10, 100, 1,000, and 10,000). The radial resistance of the very narrow junctional clefts (R_jc_) is depicted. Each smooth muscle cell is depicted by four basic units: two for the surface membrane (one upward-facing and one downward-facing) and one for each of the two end junctional membranes. To reduce complexity, the transmembrane voltage (V_m_) was recorded from only the upward-facing surface membrane.

As shown in Figure 1, there were two surface membrane units in each cell (one facing upwards and one inverted) and one unit for each of the junctional membranes. The values for the circuit parameters used (standard conditions) are listed in Table 1 for both the surface units and junctional units, and are consistent with those used previously [3,17,18].

**Table 1:** Parameter values used understandard conditions.

**Parameters**	**Surface unit**	**Junctional unit**
C_c _(pF)	5	0.5
R_K _(MΩ)	59	600
R_Ca _(MΩ)	200	2000
E_K _(mV)	-90	-90
E_Ca _(mV)	+60	+60
R_d _(MΩ)	5000	5000
C_d _(pF)	30	30
	**Common**	
R_or _(KΩ)	1.0	
R_ol _(KΩ)	1.0	
Ri (KΩ)	1000	
R_jc _(MΩ)	300	
R_bt _(KΩ)	1.0	

C_c _= Total cell capacitance

R_K _= Potassium resistance

R_Ca _= Calcium resistance

E_K _= Potassium equilibrium potential

E_Ca _= Calcium equilibrium potential

R_d _= Resistance in delay circuit

C_d _= Capacitance in delay circuit

R_or _= Radial resistance of external fluid

R_ol _= Longitudinal resistance of external fluid

R_i _= Longitudinal resistance of intracellular fluid

R_ir _= Radial resistance of intracellular fluid

R_jc _= Radial resistance of junctional cleft

R_bt _= Bundle termination resistance

**Table 2:** Black-box values for surface membraneunit and junctional membrane unit

A. Surface membrane unit			
**Black-box #1**		**Black-box #2**	

**Voltage (mV)**	**Current (nA)**	**Voltage (mV)**	**Current (nA)**

-55	-0.57	55	0
-50	-0.71	-	-
3	-1.65	-	-
10	-1.67	-10	-1.1

**B. Junctional membrane unit**			

**Black-box #1**		**Black-box #2**	

-55	-0.057	55	0
-50	-0.013	-	-
3	-0.165	-	-
10	-0.167	-10	-0.11

**Table 3:** Summary of TPT and θ values as a function of number of gap-junction channels.

		**Rbt = 1.0 KΩ**		**Rbt = 600 MΩ**	
**Number of gap-junction channels **^#^	**Rgj (MΩ)**	**TPT (ms)**	**Θ (cm/s)**	**TPT (ms)**	**Θ (cm/s)**

0	100,000	9.0*	17.8*	9.3	19.4
1	10,000	8.8*	18.2*	8.0	22.6
10	1,000	8.0	22.5	6.3	28.7
100	100	2.8	64.3	2.0	90.0
1,000	10	0.1	1800	0.04	5143
10,000	1	0	8	0	8

# Each gap-junction channel was assumed to have a conductance of 100 picosiemens (pS)

* For only 9 cells, because cell #10 failed to fire.

The smooth muscle cell was assumed to be a cylinder 200 μm long and 6 μm in diameter. The cell capacitance was assumed to be 30 pF, and the input resistance to be 20 MΩ. But to increase +dV/dt max to a value appropriate for visceral smooth muscle, the total cell capacitance was reduced 11 pF. A junctional tortuosity (interdigitation) factor of 4 was assumed for the cell junction [3]. The junctional cleft potential (V_jc_) is produced across R_jc_, the radial resistance of the narrow and tortuous junctional cleft. The junctional cleft contained two radial resistances (R_jc_) of 600 MΩ each in parallel (i.e., R_jc _= 300 MΩ). The value assigned to R_jc _reflects the thickness of the junctional gap (end-to-end) and the tortuosity factor.

The circuit used for each unit was kept as simple as possible, using only those ion channels that set the resting potential (RP) and predominate during the rising phase of the AP. The RP was -56 mV, and the overshoot potential was +9 mV (AP amplitude of 65 mV). Because the PSpice program does not have a V-dependent resistance to represent the increase in conductance for Ca^++ ^ions in smooth muscle cells during depolarization and excitation, this function was simulated by a V-controlled current source ("black-box", BB) in each of the basic circuit units. The current output of the BB, at various membrane voltages, was calculated assuming a sigmoidal relationship between membrane voltage and resistance between -50 mV and -20 mV. The appropriate values were inserted into the GTABLEs for each BB [3].

**Figure 2:**
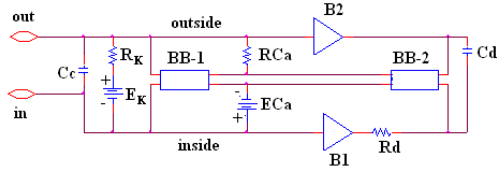
Circuit diagram for one of the basic units. The unit circuitry was the same for both the surface units and junctional units, but the values of the various components were adjusted to reflect the long surface membrane and short junctional membrane. These values are listed in Table 1. The GTABLE values for the two types of membrane were also different, and these are listed in Table 2. Note that there are two black-boxes (BB) in the basic unit, and both are in the same Ca^++ ^conductance leg of the Hodgkin-Huxley circuit. The first BB produced activation of the Ca^++^conductance, and the second BB produced deactivation of the Ca^++^channel conductance. It was necessary to produce a time delay (RC time constant) before the second BB came into play to cause deactivation. The two operational amplifiers (unity gain) depicted acted as buffers. In the K^+^leg, the K^+ ^conductance is in series with E_K_, the K^+^equilibrium potential.

Our novel approach to simulation of the entire action potential (AP) waveform was achieved by inserting a second BB into the Ca^++ ^leg of the basic unit. Thus, the first BB mimics Ca^++ ^activation, and the second BB mimics deactivation of the Ca^++^-channel conductance. The latter allowed repolarization of the AP to occur. BB-2 is connected between the outside and inside of the membrane unit, with reversed polarity compared to BB-1, as shown in Figure 2. The outputs of BB-1 and BB-2 were tied together in such a way that BB-2's output current nullifies BB-1's output current. BB-2 was activated with a delay time corresponding to the physiological delay value (i.e., to give an appropriate action potential duration (APD_50_)). The required delay time was generated using a delay element R_d _C_d _(RC time constant) (Fig. 2). At the resting potential, BB-2's output current was set to 0 nA. Once the cell has fired using BB-1's current, the potential across the input of BB-2 starts increasing with a rate corresponding to the RC time constant of the delay element. Thereby, BB-2 starts responding to the input rising voltage. Two buffer elements (unity gain operational amplifiers) were added to isolate the input terminal of BB-2 from BB-1, thus avoiding any hindrance between the two BBs.

The chain of 10 cells was assumed to be bathed in a large volume of Ringer solution connected to ground. The external resistance (R_o_) of this fluid was divided into two components: a radial resistance (R_or_) and a longitudinal resistance (R_ol_). The cells in the chain were either connected by low-resistance pathways (gap-junction channels) or not interconnected, so that transmission of excitation from one cell to the next had to rely on the electric field (EF) developed in the junctional cleft. The ends of the chains had a bundle termination resistance (R_bt_) of 1.0 KΩ to mimic the physiological condition, but was increased to 600 MΩ in some experiments to counter end-effects. R_bt _was increased to equal R_jc_.

Electrical stimulation (rectangular current pulses of 0.25 nA and 0.50 ms duration) was applied to the inside of the first cell of the chain (cell # 1). To minimize confusion, the voltage was recorded from only one surface unit (upward-facing) in each cell. Propagation velocity was calculated from the measured total propagation time (TPT) (measured as the difference between when the APs (rising phase) of the first cell and last cell crossed -20 mV) and the assumed cell length of 200 μm.

## Results

**Figure 3:**
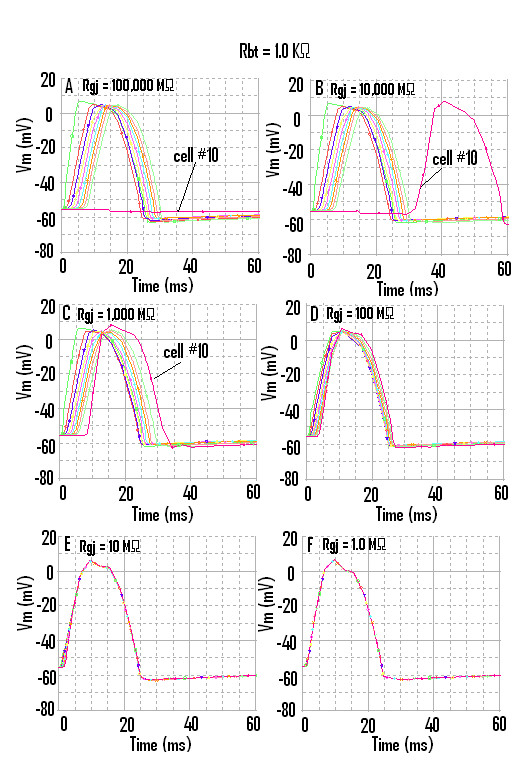
Propagation of APs simulated by PSpice along a chain of ten smooth muscle cells. The termination resistance at the ends of the chain (R_bt_) was 1.0 KΩ, similar to that for the fluid bathing the surface of the long chain. The six panels show the effect of varying the number of gj-channels from zero (panel A, R_gj _= 100,000 MΩ), to one (panel B, R_gj _= 10,000 MΩ), to 10 (panel C, R_gj _= 1,000 MΩ), to 100 (panel D, R_gj _= 100 MΩ), to 1000 (panel E, R_gj _= 10 MΩ), to 10,000 (panel F, R_gj _= 1.0 MΩ). At the R_bt _of 1.0 KΩ, the last cell in the strand (cell #10) failed to become excited (panel A), or it fired after a long delay (panel **B**), when there were no gj-channels (**A**) or only one gj-channel (**B**), and so transmission from cell to cell relied on the EF mechanism. Thus, there was a prominent end (edge) effect. In addition, the overshoot amplitude of the AP in cell #10, as well as in cell #1, was greater (**A**, **B**, **C**). Thus, end effects occurred at both ends of the strand. Note the presence of a hyperpolarizing after-potential following the repolarizing phase of the AP. When there were many gj-channels (EF), the APs of all 10 cells were superimposed, indicating extremely fast propagation velocity.

The AP waveform and propagation down the chain of 10 cells, when the strand termination resistance (R_bt_) was 1.0 KΩ, is illustrated in Figure 3. Propagation was studied with 0, 1, 10, 100, 1000 and 10,000 gj-channels traversing the cell junctions. Assuming each gj-channel has a conductance of 100 pS, these channels corresponded to a shunt resistance across each junction (R_gj_) of 100,000 MΩ, 10,000 MΩ, 1,000 MΩ, 100 MΩ, 10 MΩ, and 1.0 MΩ, respectively. The corresponding records are shown in Figure 3, panels A through F.

When there were no gj-channels (Fig. 3A), or only one channel (Fig. 3B), fast propagation still occurred, which was initiated by the EF mechanism. As can be seen, the last cell (cell #10) failed to fire (Fig. 3A). The reason for the failure of cell #10 will be evident when the next figure is discussed. In Figure 3B (1 gj-channel), cell #10 fired after a prolonged delay; this delay was reduced in Fig. 3C (10 gj-channels). When there are 1000 (Fig. 3E) or 10,000 (Fig. 3F) gj-channels, then the APs of all 10 cells are superimposed. Thus, the TPT is nearly 0 ms in these panels.

**Figure 4:**
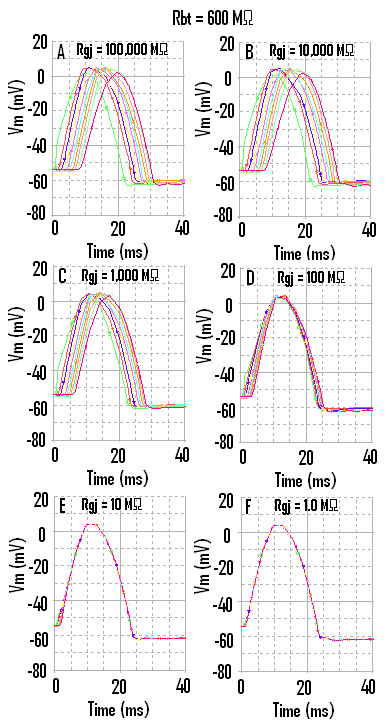
When the strand termination resistance was increased to 600 MΩ, then the last cell (cell #10) did not fail when there were no gj-channels (**A**) or only one gj-channel (B). In addition, the overshoot amplitude of the APs in cell #1 and cell #10 was smaller than in cells 2–9. Thus, the end effect now produced smaller overshoots.

Propagation down the chain when the strand termination resistance R_bt _was 600 MΩ is illustrated in Figure 4. As can be seen, all 10 cells fired an AP, even with no gj-channels (Fig. 4A) or only 1 gj-channel (Fig. 4B). Also evident in Figure 4 is the fact that the overshoot of the APs in cells #10 and #1 are smaller than in cells 2–9. When there were 1000 or 10,000 gj-channels, the APs of all 10 cells were superimposed (Fig. 4E, F). The TPT is nearly 0 msec in Fig. 4E and 4F. The APD_50 _is slightly lower at the very high R_bt _value. The peak of the AP was also more smooth (or rounded) at the high R_bt _value (e.g., compare Fig. 4F with Fig. 3F). The hyperpolarizing after-potential was about the same in the two cases.

**Figure 5:**
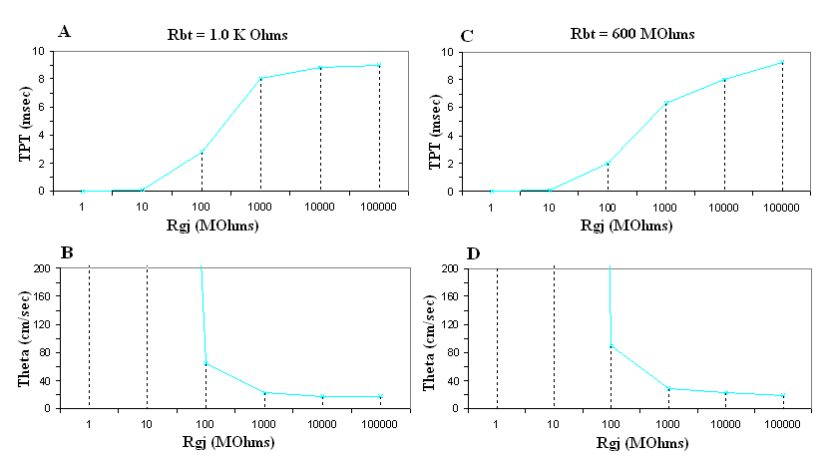
Graphic summary of the total propagation time (TPT) (**A**, **C**) and propagation velocity (θ, theta) (**B**, **D**) as a function of the shunt resistance across the 9 cell junctions (R_gj_). The data for a strand termination resistance (R_bt_) of 1.0 KΩ (A, B) and 600 MΩ (C, D) are given in separate plots. TPT was the time difference between when the AP of cell #1 and cell #10 crossed a V_m _of -20 mV. The velocity (θ) was calculated from the TPT, assuming a cell length of 200 μm. Assuming the conductance of the gj-channel is 100 pS, the R_gj_values shown of 1.0, 10, 100, 1,000, 10,000, and 100,000 MΩ correspond to the number of gj-channels of 10,000, 1,000, 100, 10, 1.0 and 0.

Figure 5 gives the graphic plots of TPT and θ as a function of R_gj _for both R_bt _of 1.0 KΩ (Fig. 5A, B) and R_bt _of 600 MΩ (Fig. 5C, D). As shown, as R_gj _decreases (reflecting more and more gj-channels), the TPT decreases and θ increases, for both low (1.0 KΩ) and high (600 MΩ) values of R_bt_. These data are also summarized in Table 3, to facilitate comparison of the quantitative numbers.

## Discussion

The present results demonstrate that it is now possible to simulate the entire AP, both the rising phase and the falling phase, in PSpice simulations. Since a second BB (BB-2) could not be inserted into the K^+^leg of the basic Hodgkin-Huxley membrane units because of instability of the PSpice program, the problem was solved by inserting the BB-2 into the Ca^++ ^leg to produce deactivation of the Ca^++ ^conductance turned on by the first BB (BB-1). BB-2 supplied current in the opposite polarity of that supplied by BB-1, giving a net current of zero. BB-2 supplied its current after a time delay provided by an RC time constant. Thus, BB-1 produced the rising phase of the AP, and BB-2 produced the falling (repolarizing) phase. Therefore, it is now possible to use the PSpice program to study propagation of complete APs in single strands and in two-dimensional sheets (parallel strands).

The after-hyperpolarization that was produced following the "spike" of the AP, (e.g., see Fig. 3A) is equivalent to the physiological hyperpolarizing after-potential seen in smooth muscle cells. It is due to a "feed-forward" mechanism. When BB-2 is supplying its opposing current and producing partial repolarization of the AP, the current supplied by BB-1 starts to decrease because the GTABLE is reversible [22]. Therefore, BB-2 supplies an excess of current, causing the after-hyperpolarization.

The data showed that, when R_bt _was 1.0 KΩ, cell #10 failed to fire when the transmission of excitation from cell to cell depended on the EF mechanism (Fig. 3A). It was previously shown that, with the EF mechanism, the distal portion of the cell reached threshold and fired before the proximal portion [3,5]. Increasing R_bt _to 600 MΩ enabled the firing of cell #10 when the EF mechanism was dominant (Fig. 4A).

When cell #10 failed to fire, there was no passive partial depolarization produced due to electrotonic spread of current (Fig. 3A). This underscores the fact that there were no low-resistance connections or gj-channels between cells 9 and 10 or between the other cells. Yet propagation occurred at a fast velocity, enabled entirely by the EF mechanism. When there were 1000 or 10,000 gj-channels, the propagation velocity became extremely fast, i.e., non-physiological.

There were substantial end (edge) effects at both ends of the strand. It can be seen in Figure 3A–C, that the overshoot of the APs (hence that AP amplitude) was greater in cell #1 and cell #10 compared to those in cells 2 – 9. In contrast, when R_bt _was 600 MΩ, the overshoot of the APs in cell #1 and cell #10 was smaller than in cells 2 – 9 (Fig. 4 A-B). Thus, the edge effect caused the AP amplitude in the end two cells to be either greater or smaller, depending on the value of R_bt _. We are unable, at this time, to compare the end-effects observed in the PSpice simulations with those reported for biological tissues.

In summary, it was now possible to produce complete APs in PSpice simulation by placing a second BB (BB-2) into the Ca^++ ^leg of the basic membrane units which supplied an opposing current after a short time delay. This opposing current essentially deactivated the inward Ca^++ ^current that was supplied by BB-1. The single strand of 10 cells exhibited prominent edge effects at the two ends of the strand. As previously reported, propagation in smooth muscle occurred at physiological velocities when no gj-channels, one gj-channel or 10 gj-channels was present. The propagation velocity became very fast and non-physiological when 1000 or 10,000 gj-channels were present. A large R_bt _value enabled the firing of cell #10 by the EF mechanism.
